# Utility of Bruton's Tyrosine Kinase Inhibitors in Light Chain Amyloidosis Caused by Lymphoplasmacytic Lymphoma (Waldenström's Macroglobulinemia)

**DOI:** 10.1155/2022/1182384

**Published:** 2022-01-19

**Authors:** Maroun Bou Zerdan, Jason Valent, Maria Julia Diacovo, Karl Theil, Chakra P. Chaulagain

**Affiliations:** ^1^Department of Hematology-Oncology, Myeloma and Amyloidosis Program, Maroone Cancer Center, Cleveland Clinic Florida, Weston, FL, USA; ^2^Multiple Myeloma Program, Taussig Cancer Center, Cleveland Clinic, Cleveland, OH, USA; ^3^Department of Pathology, Cleveland Clinic Florida, Weston, FL, USA; ^4^Department of Laboratory Medicine, Cleveland Clinic, Cleveland, OH, USA

## Abstract

Of the variety of immunoglobulin related amyloidosis (AL), immunoglobulin M (IgM) related AL represents only 6 to 10% of affected patients, and the majority of these cases are associated with underlying non-Hodgkin's Lymphoma including Waldenström's macroglobulinemia (WM). Ibrutinib, acalabrutinib, and zanubrutinib are Bruton tyrosine kinase (BTK) inhibitors approved for certain indolent B cell non-Hodgkin's lymphoma (NHL). BTK is a nonreceptor kinase involved in B-cell survival, proliferation, and interaction with the microenvironment. We retrospectively evaluated the tolerability and effectiveness of BTK inhibitors ibrutinib and acalabrutinib therapy in (*n* = 4) patients with IgM-related AL amyloidosis with underlying WM. Treatment was well tolerated with both hematologic and organ response in patients with AL amyloidosis in the setting of WM. Atrial fibrillation led to the discontinuation of ibrutinib in one patient, and acalabrutinib caused significant thumb hematoma needing dose reduction in another patient. All patients evaluated had the MYD88 mutation. This may explain the good response to BTK inhibitors therapy in our series. BTK inhibitors should be further investigated in larger prospective studies for treatment of AL amyloidosis in patients with lymphoplasmacytic lymphoma/WM.

## 1. Introduction

Previously referred to as primary amyloidosis, immunoglobulin light chain amyloidosis (AL) is a clonal plasma cell proliferative disorder characterized by tissue deposits of immunoglobulin light chain fragments, leading to organ dysfunction [1]. AL amyloidosis is characterized by extracellular deposition of insoluble fibrils composed of misfolded molecules of immunoglobulin light chains [2]. The variable region of *λ* light chains constitutes the latter in approximately 75% of cases and К in the remainder [3, 4]. Out of the variety of AL amyloidosis, immunoglobulin M (IgM) related AL represents around only 6–10% of affected patients, and 54% of these cases are related to underlying non-Hodgkin's lymphoma including Waldenström's macroglobulinemia, and the remaining cases are related to pure clonal plasma cell neoplasm [5–7]. Evaluation of IgM AL in the setting of WM requires testing for *CXCR4* and *MYD88* mutation status due to their prognostic and therapeutic implications [8]. As first reported by Chakraborty et al., 71% (10 out of 14) of patients harbored an *MYD88* mutation; these patients had a different clinical outcome compared to others with an *MYD88* wild type [9]. This mutation is found in 90% of cases of WM/lymphoplasmacytic lymphoma, and the *MYD88* L265P mutation is associated with a higher risk of disease progression but a better response to Bruton's tyrosine kinase inhibitors (BTKIs) therapy compared to others with wild-type *MYD88*. WM patients with *CXCR4* mutations have decreased responses to BTKIs; in other words, responses to BTKIs are higher and deeper among those with mutated *MYD88* and wild-type *CXCR4* [10, 11].

The treatment protocols of MM have been beneficial in non-IgM-related AL, but it is in IgM-related AL where more work is needed [2]. Considering the clinically indolent nature of WM, it is necessary to point out that in cases of AL due to WM, the AL is the main cause of morbidity and mortality, and therefore, a WM patient with an AL needs immediate therapy even if the WM is asymptomatic at the time of presentation [2]. Although protocols for MM therapy have been successfully implemented for the treatment of non-IgM-related AL, experience with protocols used for WM is scarce in the treatment of IgM-related AL. Palladini et al. reported that 78% (7 patients) were able to achieve a hematologic response after two cycles of RBDex (rituximab, bortezomib, and dexamethasone), including 3 patients who had refractory disease to prior therapy with rituximab [12]. Autologous hematopoietic stem cell transplantation (HSCT) in 12 patients showed that the organ response rate was 67% and the hematologic response was 89% [13]. Last, in a French nationwide retrospective study, purine analogs induced a hematologic response in 73% with a complete response in 9% and an organ response in 55% [14].

## 2. Discussion and Patient Series

Ibrutinib has been approved for the treatment of chronic lymphocytic lymphoma/small lymphocytic lymphoma (CLL/SLL), mantle cell lymphoma (MCL), and WM. Ibrutinib, acalabrutinib, and zanubrutinib have all been approved for the treatment of MCL patients who have received at least 1 prior therapy [5].

### 2.1. Materials, Methods, and Findings

**Figure 1:**
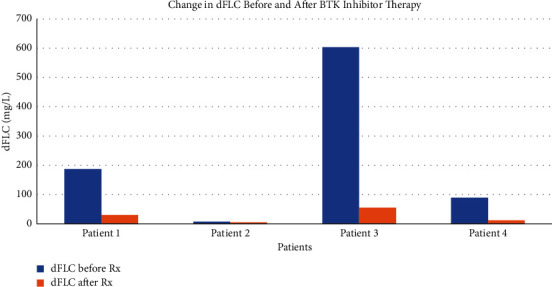
Change in difference of free light chains before and after BTK inhibitor therapy. dFLC: difference in free light chain and Rx: therapy.

**Figure 2:**
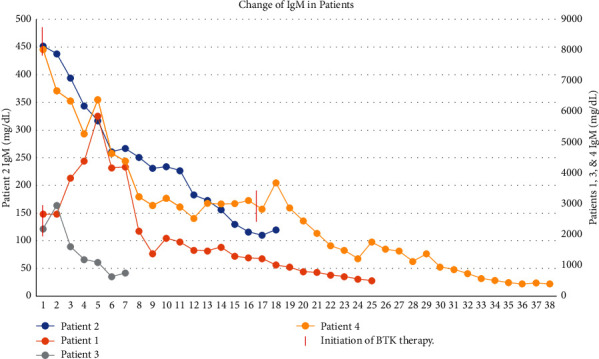
Change of IgM in patients.

In this Institutional Review Board approved protocol of Cleveland Clinic, we retrospectively evaluated the effects of BTK inhibitors ibrutinib and acalabrutinib therapy in patients (*n* = 4) with IgM-related AL amyloidosis due to underlying WM. The patients' characteristics are presented in Table 1. Patients were equally split between two males and two females with a median age of 73 (range 60–91) when BTKI therapy was started. The *MYD88* L265P mutation was present in all patients. The *CXCR4* mutational status was not evaluated. Three out of four patients received BTKI as the first line treatment, and one received BTKI as the second line. The median IgM before BTKI therapy started was 3521.75 mg/dL (range 40–230 mg/dL), and the median M-protein spike was 2.3 g/dL. Two patients received ibrutinib, and the other two received acalabrutinib. The hematologic and organ responses were assessed based on previously published criteria [15]. All 4 patients had excellent durable hematologic responses to BTKI therapy with two patients achieving VGPR and the other two securing a CR. All patients had symptomatic improvement and organ response (Table 1). The difference in free light chains is depicted in Figure 1, and the decrease in IgM for each patient over time during the therapy is seen in Figure 2.

#### 2.1.1. Patient 1

**Figure 3:**
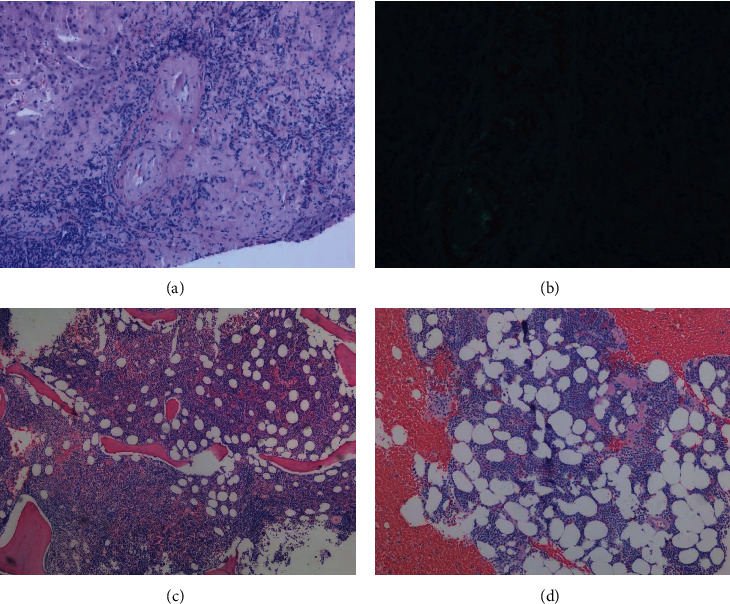
(a) Liver needle biopsy, H and E 10x: glassy light eosinophilic material deposited among hepatocytes and on vessel walls. (b) Liver needle biopsy, Congo red stain 20X: polarization of Congo red shows apple-green birefringence, most prominently around the vessel wall. (c) BM H and E 5x: diffuse involvement by small atypical lymphoid cells and rare plasma cells (60% of cellularity). (d) BM clot H and E 10x: bone marrow spicule diffusely involved by small atypical lymphoid cells.

**Figure 4:**
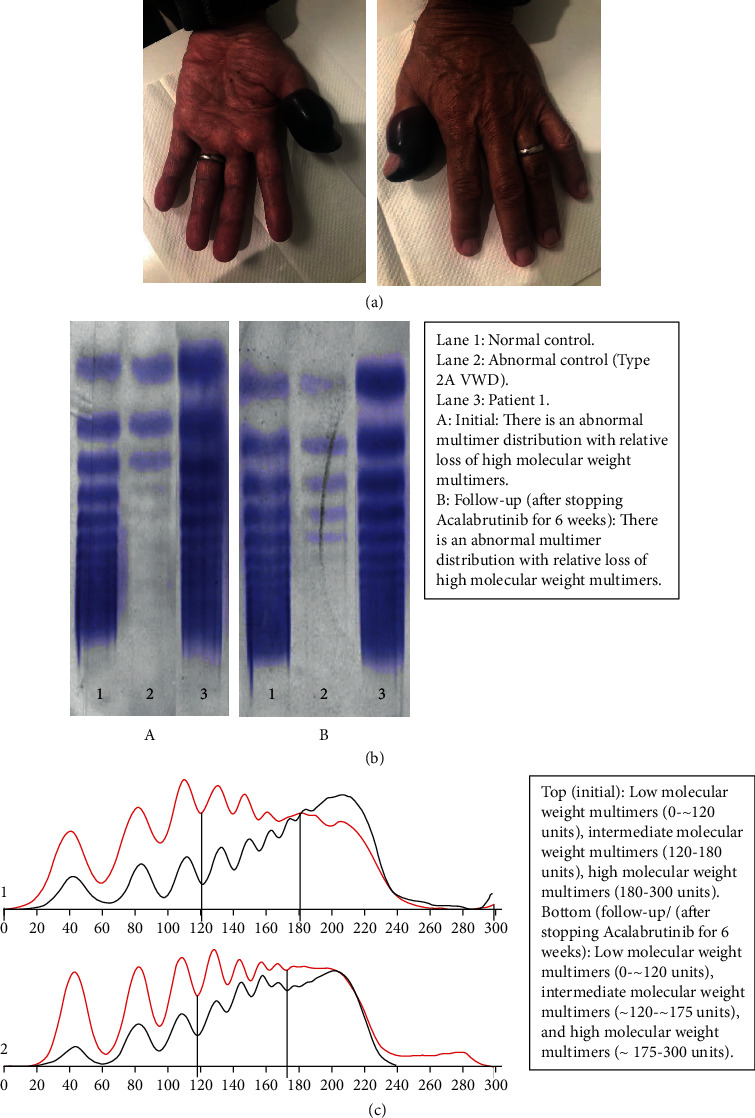
(a) Thumb hematoma. (b) Multimeric analysis of von Willebrand factor was performed by an agarose gel electrophoresis followed by immunofixation with antivon Willebrand factor antiserum. (c) Densitometry scan of an agarose gel following immunofixation. There is an abnormal multimer distribution with a relative loss of high molecular weight multimers (patient: blue line; normal control: black line).

Patient 1 is a male with a history of WM for 14 years, managed with hematologic monitoring without therapy, presented in 2020 with 22 lbs. of weight loss over a period of two years along with poor appetite, hoarseness of the voice, dyspnea with minimal exertion, early satiety, abdominal bloating, and right upper quadrant abdominal fullness and pain. On physical examination, he had hepatomegaly of 10 cm below the right costal margin in the right midclavicular line. Further workup revealed cholelithiasis and his symptoms were attributed to cholelithiasis which prompted a laparoscopic cholecystectomy which was aborted due to a cirrhotic-appearing liver intraoperatively. A liver biopsy demonstrated extensive amyloid deposits (Figure 3(a)) having an apple-green birefringence when stained with Congo red and observed under polarized light (Figure 3(b)). A *MYD88*-positive lymphoplasmacytic lymphoma was confirmed by a bone marrow biopsy involving ∼50% of the bone marrow cellularity (Figure 3(c)). An echocardiogram showed a left ventricular ejection fraction of 40%, grade III diastolic dysfunction, NT-proBNP was elevated to 4928 (range 0–450 pg/mL) and cardiac troponin T was elevated to 0.042 (range 0.000–0.029 ng/mL). This led to an endomyocardial biopsy which also revealed amyloidosis. Liquid chromatography-mass spectrometry (LC-MS) analysis of both the liver and heart biopsies confirmed that the main amyloidogenic component was the К immunoglobulin light chain. He was started on rituximab and acalabrutinib and the therapy was complicated with rituximab flare (after the first dose of rituximab) and hyperviscosity-related epistaxis that responded well to plasma exchange, and he was able to continue the therapy with a continued sustained antilymphoma response over the next several months (Figures 1 and 2). Interestingly, there was a 6 cm decrease in hepatomegaly along with >50% decline in the alkaline phosphatase level after a year of therapy with rituximab and acalabrutinib. About nine months into the acalabrutinib therapy, the therapy was complicated with an episode of left subungual hematoma (Figure 4(a)) following a trivial nail bed trauma. Acalabrutinib was held and hematoma was evacuated. At that time, platelet count, prothrombin time (PT), partial thromboplastin time (PTT), and international normalized ratio (INR), factor VIII, factor IX, and von Willebrand antigen levels and function were all normal (Table 2). A von Willebrand multimer analysis revealed abnormal multimer distribution with relative loss of high molecular weight multimers (Figures 4(b) and 4(c) and Table 2). Finally, acalabrutinib was resumed at a 50% dose reduction (100 mg/day) after six weeks which was tolerated without recurrent bleeding events. At the time of writing this report, he continues to maintain a complete hematologic response along with cardiac and hepatic responses and enjoys a good quality of life.

#### 2.1.2. Patient 2

Patient 2 is a woman who presented in 2019 with a history of chronic kidney disease (CKD) for the past two years. Evaluation revealed elevated creatinine of 2.41 mg/dL (range 0.58–0.96 mg/dL), with an estimated glomerular filtration rate of 20 mL/min/1.73 m^2^. A diagnosis of stage IV CKD was made. The complete blood count was notable for normocytic normochromic anemia with a hemoglobin level (Hb) of 10.5 g/dL (range 11.5–15.5 g/dL). Further workup revealed IgM lambda monoclonal gammopathy without significant proteinuria (Table 1). A bone marrow biopsy confirmed *MYD88*-positive lymphoplasmacytic lymphoma involving ∼50% of the bone marrow cellularity (Figure 3(d)). A monoclonal gammopathy of renal significance (MGRS) was suspected, and a kidney biopsy was recommended. A renal biopsy revealed AL amyloidosis involving the interstitium, vessel walls, and glomeruli. No cardiac or liver involvement was identified. The mild elevation of NT-proBNP likely represents decreased clearance due to elevated creatinine. The patient was started on Rituximab (375 mg/m^2^ weekly × 4 then every 2 months × 2 years) and ibrutinib (420 mg daily) for AL amyloidosis with advanced CKD. Throughout her treatment, the patient tolerated ibrutinib and only reported occasional bruises on her forearms. Approximately 14 months into the treatment, she has achieved a complete hematologic response without progression of chronic kidney disease (CKD) and without the need for dialysis.

#### 2.1.3. Patient 3

Patient 3 is a woman who presented with refractory congestive heart failure (CHF), transfusion dependent anemia, stage IV CKD, and failure to thrive (Table 1). Anemia works up to a bone marrow biopsy which revealed a *MYD88*-positive lymphoplasmacytic lymphoma involving ∼90% of the bone marrow cellularity with minimal residual hematopoiesis. She had severe infusion reactions to one dose of rituximab followed by a dose of bortezomib; a single dose of both drugs led to fluid overload/CHF exacerbation and several days of hospitalization. Acalabrutinib was started at a 50% reduced dose (100 mg daily) adjusted for advanced age and multiple comorbidities. The patient tolerated the regimen, and at 9 months into the therapy, has achieved a very good partial response (VGPR) along with a cardiac response and remains dialysis independent.

#### 2.1.4. Patient 4

Patient 4 is a male patient who presented in 2016 having a one-month history of dyspnea. Upon imaging, there was extensive, confluent retroperitoneal, pelvic, and left groin adenopathy, and a lymph node biopsy identified a CD20+ small B-cell neoplasm (*MYD88*-positive) with plasmacytic differentiation and marked AL amyloid deposition. An echocardiogram and cardiac biomarkers were consistent with cardiac amyloidosis (Table 1). The decision was made to start him on a regimen containing bortezomib, dexamethasone, and rituximab (BDR), and he achieved a partial response after 5 months of therapy. A decision was made to switch therapy to ibrutinib 420 mg daily along with an overlap of rituximab every 2 months. After 10 months of ibrutinib and rituximab therapy, the patient's hematologic response improved to VGPR [16], but due to a new onset of atrial fibrillation, ibrutinib had to be discontinued. A CT scan at that time revealed a growing nodal mass in the retroperitoneum despite sustained hematologic VGPR and cardiac biomarker response. Due to the concern for nodal progression, he was started on bendamustine and rituximab to curtail the growing nodal disease, and he completed six cycles with a hematologic CR and continued cardiac response. Last, after 10 months of observation, the patient started to have both serologic and nodal relapse and evidence of cardiac and renal progression. He was enrolled in a clinical trial containing bortezomib, Cytoxan, dexamethasone (CyborD), and an experimental monoclonal antibody against amyloid fibrils. The patient is tolerating therapy well with no therapy-related adverse effects at the time of this writing.

## 3. Discussion and Conclusion

Here we report on four patients treated with BTKIs (2 patients with ibrutinib and 2 with acalabrutinib) in the earlier lines of therapy (3 first line and 1 second line) for IgM-related AL amyloidosis due to *MYD88* mutated WM. Our patients tolerated BTKIs well and had excellent hematologic responses as well as organ responses (Table 1 and Figures 1 and 2). Our patients were not optimal candidates for cytotoxic chemotherapy due to advanced AL cardiomyopathy, advanced nephropathy, or extreme age; therefore, we sought to treat them with BTKIs given their better toxicity profile and tolerability. Patients with underlying AL cardiomyopathy were treated with acalabrutinib, and those without AL cardiomyopathy received ibrutinib. One patient (patient 3) who received ibrutinib in the setting of AL cardiomyopathy had to discontinue ibrutinib due to new onset atrial fibrillation. All BTKIs have similar efficacies, but they differ in their side effect profiles. For example, acalabrutinib causes less atrial fibrillations when compared to ibrutinib and may be better tolerated by patients with underlying cardiac conditions. Studies have shown that the rates of grade 3 atrial fibrillations with ibrutinib are higher than those reported with acalabrutinib (6% with ibrutinib vs. 3% with acalabrutinib) [17]. In another phase III clinical trial comparing acalabrutinib and ibrutinib in chronic lymphocytic leukemia (CLL), all-grade atrial fibrillation/atrial flutter incidence was significantly lower with acalabrutinib versus ibrutinib (9.4% v 16.0%; *P*=0.02) [18].

Similarly, a randomized phase 3 trial of zanubrutinib vs. ibrutinib in symptomatic Waldenström's macroglobulinemia (ASPEN study) reported significantly higher atrial fibrillation/flutter for ibrutinib than zanubrutinib (15.3% vs. 2%, respectively; *P* < 0.05) [19]. These data suggest that patients with underlying cardiac conditions can probably tolerate acalbabrutinib or zanubrutinib better than ibrutinib. Our two patients with underlying cardiac AL amyloidosis received acalabrutinib without any cardiac arrhythmias, but one patient with AL cardiomyopathy who was on ibrutinib had atrial fibrillation leading to discontinuation.

Clinically significant bleeding events are more common with patients on ibrutinib accounting for 5% of patients on ibrutinib as compared to 3% in patients treated with acalabrutinib [20]. One patient in our series had subungual hematoma after a minor trauma. The relative loss of high molecular weight MW multimers found in our patient can be seen in WM, where in some cases, loss of the high MW multimers mimics a type 2b von Willebrand disease (VWD) pattern in acquired VWD [21–23]. We are monitoring this patient to see if the relative loss of high MW multimers goes away as the IgM monoclonal protein decreases. We did not find that acalabrutinib contributed to the relative loss of high MW multimers based on multimeric analysis performed at the time of the hematoma and on a repeat test six weeks after suspension of acalabrutinib therapy (Figures 4(b) and 4(c) and Table 2). This observation indicates that the type 2 b VWD pattern observed in this patient is due to underlying WM and not due to acalabrutinib therapy.

IgM-related AL amyloidosis has been previously investigated by Sissoko et al. at the Boston University Amyloidosis Center. The organ involvement rates in 95 patients were 51%, 40%, 25%, and 17% for the kidney, heart, lymph nodes, and gastrointestinal tract, respectively. Among the 46 treated patients whose responses were analyzed, patients with a hematologic response had a superior 5-year survival rate than nonresponders (79.2 ± 8.5% versus 41 ± 14.9%) [24]. In another study involving 103 patients at the National Amyloidosis Center (London, United Kingdom), renal, cardiac, and lymph node involvement was present in 53%, 35%, and 21% of patients, respectively [25]. Patients with lymph node involvement did not show any nodal improvement to therapy. Last, in a retrospective study involving 250 patients diagnosed with IgM-related AL amyloidosis from three European amyloidosis centers, it was suggested to combine abnormal NT-proBNP and troponin T with liver involvement and the presence of neuropathy as a risk model. Based on the risk stratification model, the median overall survival of patients with none, one, or two or more abnormal factors was 90, 33, and 16 months, respectively [6].

Management of heavily pretreated refractory/relapsed patients with IgM-related AL amyloidosis with ibrutinib was found to be difficult in a small (*n* = 8) retrospective series with only one patient achieving VGPR [2]. However, there is a paucity of data regarding the use of BTKI (ibrutinib or acalabrutinib) in the earlier lines of therapy (first- or second-line setting) in patients with IgM-related AL amyloidosis due to WM. When ibrutinib is used in the earlier line of therapy, a favorable response is possible in patients with WM without AL amyloidosis. For example, Treon et al. reported a 5-year overall survival rate of 87% with a few reported adverse events for 63 WM patients with two median prior therapies [26]. In this series, the patients with mutated *MYD88* and wild-type *CXCR4* had faster and deeper responses and better progression-free survival at 5 years.

Our study has the limitation for being a small retrospective single institutional study but does provide evidence that careful patient selection can potentially lead to the successful use of ibrutinib and acalabrutinib for the treatment of systemic AL amyloidosis due to IgM-related AL amyloidosis secondary to *MYD88* mutated WM. Furthermore, *CXCR4* mutation status was not available in our series, and it should be noted that WM patients with *CXCR4* mutation tend to have an inferior response to BTK inhibitors. For this reason, BTK inhibitor therapy should not be considered the frontline therapy option in WM patients who are MYD88 wild-type and CXCR4 mutated. While selecting therapy for the treatment of newly diagnosed AL in the setting of WM, it is also important to note that frontline BTK inhibitor therapy is usually a long-term commitment and can be associated with side effects over time, as opposed to a fixed duration of 6 months or less treatment with chemoimmunotherapy such as bendamustine and rituximab (B-R). In addition, the response to chemoimmunotherapy such as B-R is not dependent on the CXCR4 and MYD88 status and may be a desirable option for patients if the mutational status is unknown. In WM, the IgM rebound phenomenon has been observed in >50% (14 out of 25 patients) of patients who discontinue ibrutinib abruptly leading to symptomatic hyperviscosity in one out of 25 such patients in median follow-up of 2 months [27]. Clinicians should be aware of this phenomenon and follow patients closely clinically. Two of our patients discontinued BTK inhibitor therapy without observation of this phenomenon (patient 1 stopped for ∼6 weeks due to subungual hematoma and resumed, patient 4 discontinued permanently due to atrial fibrillation). Options, risk, benefits, and alternatives should be discussed with patients in making shared clinical decisions. Larger multicenter prospective studies are needed for further clarity in this regard. Future research direction can also be the inclusion of *CXCR4* inhibitors in the treatment of IgM AL due to WM. The use of *CXCR4* inhibitors (NCT03225716 and NCT04274738) with ibrutinib is currently under investigation in patients with *CXCR4* mutated WM and may provide discernments into overcoming the adverse outcomes of this mutation in patients with WM treated with BTKIs. Prospective, randomized studies against other commonly used treatment options, such as bendamustine and rituximab, other BTKIs (such as zanubrutinib), and combinations that include *CXCR4* or BCL2 inhibitors (e.g., Venetoclax), are needed to further define the optimal use, timing, and sequencing of using BTKIs in the management of IgM-related AL amyloidosis.

## Figures and Tables

**Table 1:** Patient characteristics.

Clinical characteristics	Patient 1	Patient 2	Patient 3	Patient 4
Age/sex	70s/M	60s/F	90s/F	60s/M
Initiation of antilymphoma treatment	First line: rituximab and acalabrutinib	First line: ibrutinib and rituximab	Acalabrutinib	1st line: VDR 2nd line: ibrutinib + rituximab
Clinical presentation leading to the diagnosis	Symptomatic hepatomegaly, diastolic CHF	Stage IV CKD, anemia, no significant proteinuria	Transfusion dependent anemia, CHF, stage IV CKD	Dyspnea on exertion, lymphadenopathy
Circulating monoclonal protein (g/dL)	IgM kappa, M spike 2.6	IgM lambda, M spike 0.4	IgM kappa, M spike 2.2	IgM lambda, M spike 4
IgM (40–230 mg/dL)	2666	452	2949	8020
Serum-free kappa (3.30–19.40 mg/L)	205	30	705	7
Serum-free lambda (5.7–26.3 mg/L)	3	39	101	170
Kappa to lambda ratio	68	0.81	7	0.04
dFLC at presentation	202	9	604	163
Troponin T (0.000–0.029 ng/mL)	0.042	Not tested	0.1	0.047
NT-proBNP (0–450 pg/mL)	4928	650	10500	2300
Cardiac modified Mayo stage (2015)	IIIa	N/A	IIIb	IIIa
Serum albumin (3.6–5.1 g/dL)	4.2	3.9	3.1	3.9
Creatinine (0.58–0.96 mg/dL)	1	2.41	2.5	
Cholesterol (normal is <200 mg/dL)	182	198	185	144
Alkaline phosphatase (33–130 U/L)	407	99	75	
24-hour urinary protein (normal is <200 mg)	150	191	99	130
ECHO findings	Grade III left ventricular diastolic dysfunction, LVEF 48%, mild upper septal left ventricular hypertrophy	Normal LV and RV, LVEF 58%, grade I left ventricular diastolic dysfunction	Grade II left ventricular diastolic dysfunction, LVEF 28%, there is mild upper septal left ventricular hypertrophy	Grade III left ventricular diastolic dysfunction, LVEF 57%, RVH and LVH, advanced cardiac amyloidosis with restrictive physiology, sparkling granular appearance, and apical sparing
Tissue biopsy confirming the diagnosis of AL amyloidosis	Liver, bone marrow, and cardiac biopsy	Renal biopsy	Bone marrow biopsy	Lymph node biopsy showing both LPL and amyloid
LC-MS/MS analysis	The main amyloidogenic component is kappa immunoglobulin light chains	The main amyloidogenic component is lambda immunoglobulin light chains	Amyloid type confirmed with immunohistochemistry	Amyloid type confirmed with immunohistochemistry
Bone marrow biopsy findings	40–50% involvement by LPL, amyloid +	50% involvement by LPL, amyloid −	90% involvement by LPL, gain of chromosomes 4 and 18	Not done
MYD88 status	Mutated	Mutated	Mutated	Mutated
Complications during treatment with BTK-I and rituximab	Rituximab flare, thumb hematoma (Figure 4) leading to 50% dose reduction of acalabrutinib	None	None	Atrial fibrillation leading to discontinuation of ibrutinib
Antilymphoma therapy prior to initiating BTK inhibitor-based regimen	None	None	Intolerance to rituximab and bortezomib	First line (11/2016–3/2017): VDR with PR 2nd line (4/2017–12/2017): ibrutinib + rituximab with VGPR 3rd line (1/2018–6/2018): BR with stable disease 4th line: antiamyloid fibril MAB + CyBorD in a clinical trial with VGPR
Best hematologic response/outcome with BTK inhibitor therapy/time to response	VGPR with hepatic and cardiac response/8 months	CR/12 months	VGPR/10 months	CR/9 months
Organ response/time to response	Cardiac/hepatic response/6 months	Stable disease without renal progression to date	Cardiac response/6 months Stable disease without renal progression	Cardiac response/6 months

BR: bendamustine and rituximab, BTK-I=Bruton's tyrosine kinase inhibitor, CKD: chronic kidney disease, CHF: congestive heart failure, dFLC = difference in free light chain levels, CR: complete response, LC-MS/MS: liquid chromatography with tandem mass spectrometry, CyborD: cyclophosphamide, bortezomib, and dexamethasone, LPL: lymphoplasmacytic lymphoma, VGPR: very good partial response, VDR: bortezomib, lenalidomide, and dexamethasone, LV = left ventricle, RV = right ventricle, LVEF = left ventricular ejection fraction, LVH = left ventricular hypertrophy, RVH = right ventricular hypertrophy, MAB = monoclonal antibody, and ECHO = echocardiogram.

**Table 2:** Coagulation parameters of patient 1 in the setting of acalabrutinib-related subungual hematoma.

Parameters	Reference range	Initial	Follow-up (6 weeks after holding acalabrutinib)
PT	9.7–13.0 sec	12.5	12.5
APTT	23.0–32.4 sec	31.6	31.1
Platelet count	150–400 k/uL	171	122
Factor VIII	50–173%	135	106
VWF: Ag	50–173%	162	126
VWF: RCo	42–146%	76	76
VWF: CB	41–161%	91	72
FVIII/VWF ratio	>0.4	0.8	0.8
RCo/VWF ratio	>0.4	0.5	0.6
CB/VWF ratio	>0.5	0.6	0.6
Multimeric analysis			
Low MW multimers	8.9–23.1%	41.1	34.7
Intermediate MW multimers	21.6–38.9%	34.6	32.5
High MW multimers	39.9–68.3%	24.3	32.8
IgM *κ* monoclonal protein	0 g/dL	0.41	0.33
IgM	40–230 mg/dL	638	505

vWF: von Willebrand factor, Ag: antigen, RCo: ristocetin cofactor, CB: collagen binding, and MW: molecular weight.

## Data Availability

Data supporting this research article are available from the corresponding author upon reasonable request.
